# Correction: Synthesis and functional studies of self-adjuvanting multicomponent anti-HER2 cancer vaccines

**DOI:** 10.1039/d1ra90163j

**Published:** 2021-11-16

**Authors:** Qi Feng, Xiaoyue Yu, Yixue Wang, Shiyang Li, Yang Yang

**Affiliations:** Department of Nephrology, The First Affiliated Hospital of Zhengzhou University Zhengzhou 450052 P. R. China fengqi2019@zzu.edu.cn; Research Institute of Nephrology, Zhengzhou University Zhengzhou 450052 P. R. China; Henan Province Research Center for Kidney Disease Zhengzhou 450052 P. R. China; Key Laboratory of Precision Diagnosis and Treatment for Chronic Kidney Disease in Henan Province Zhengzhou 450052 P. R. China; Department of Cardiovascular Medicine, The First Affiliated Hospital of Zhengzhou University Zhengzhou 450052 P. R. China; Clinical Systems Biology Laboratories, The First Affiliated Hospital of Zhengzhou University Zhengzhou 450052 P. R. China yangyangbio@163.com

## Abstract

Correction for ‘Synthesis and functional studies of self-adjuvanting multicomponent anti-HER2 cancer vaccines’ by Qi Feng *et al.*, *RSC Adv.*, 2021, **11**, 33814–33822, DOI: 10.1039/D1RA06146A.

The authors regret that the authors were affiliated with the incorrect institutions in the original manuscript. The corrected author list and affiliations are as shown above.

The Royal Society of Chemistry apologises for these errors and any consequent inconvenience to authors and readers.

## Supplementary Material

